# Force Metrics During the Boyle–Davis Gag in Children Undergoing Adenotonsillectomy

**DOI:** 10.3390/children13030353

**Published:** 2026-02-28

**Authors:** Enriqueta Arevalo Asensio, Anelise Schifino Wolmeister, Thomas Engelhardt, Sam J. Daniel, Samuel D. F. Wasserman, Gianluca Bertolizio

**Affiliations:** 1 Division of Pediatric Anesthesia, Montreal Children’s Hospital, McGill University Health Centre, Montreal, QC H4A 3J1, Canada; enriqueta.arevaloasensio@mail.mcgill.ca (E.A.A.); thomas.engelhardt@mcgill.ca (T.E.); samuel.wasserman@rimuhc.ca (S.D.F.W.); 2 Research Institute, McGill University Health Centre, Montreal, QC H4A 3J1, Canada; 3 Department of Pediatric Surgery, Montreal, Children’s Hospital, McGill University, Montreal, QC H4A 3J1, Canada; sam.j.daniel@mcgill.ca; 4 Department of Otolaryngology-Head and Neck Surgery, Montreal Children’s Hospital, McGill University, Montreal, QC H4A 3J1, Canada

**Keywords:** force metrics, pediatric patients, oropharyngeal suspension, force sensor, gag suspension, postoperative pain, postoperative emergence delirium

## Abstract

**Highlights:**

**What are the main findings?**

- There is no association between intraoperative force metrics and opioid requirements, emergence delirium, or hypoactive delirium following adenotonsillectomy in pediatric patients.
- Suspension time is weakly and inversely associated with the severity of postoperative pain.

**What are the implications of the main findings?**

- This study provides initial reference values of force measurements of the Boyle–Davis Gag suspension during adenotonsillectomy in children.
- Routine monitoring of force during Boyle–Davis Gag suspension may not be useful in decreasing postoperative opioid consumption and pain in children.

**Abstract:**

**Background**: In adults, the forces generated during Boyle–Davis Gag suspension correlate with postoperative pain, but no data are available in pediatrics. This study investigates the force metrics and postoperative opioid consumption, pain, emergence delirium (ED), and hypoactive delirium in children. **Methods**: Children undergoing elective partial or total adenotonsillectomy or adenoidectomy were enrolled. Intraoperative maximum and average forces, suspension time, total impulse (area under the curve of force vs. time), and postoperative opioid consumption, pain, ED, and hypoactive delirium were assessed. **Results**: Data from 43 children were analyzed. Force metrics were not associated with postoperative opioid consumption, ED, or hypoactive delirium. Compared to no pain, total impulse decreased with mild (mean difference 2.3 kN·s; 95% CI, 3.8 to 4.2; *p* = 0.02), moderate (mean difference 2.8 kN·s; 95% CI, 5.4 to 3.9; *p* = 0.011), and severe pain (mean difference 2.3 kN·s; 95% CI, 7.6 to 3.9; *p* = 0.005). Suspension time was negatively correlated with pain score (r = −0.32, *p* = 0.041). **Conclusions**: The force metrics are low and not associated with opioid consumption, ED, or hypoactive delirium. Suspension correlates weakly with postoperative pain in children.

## 1. Introduction

Tonsillectomy and adenoidectomy are among the most common surgeries performed in children, with more than 500,000 procedures carried out annually in North America [1].

Optimal surgical exposure is obtained by combining head and neck extension, adequate mouth opening, and laryngeal suspension. The Boyle–Davis Gag suspension mouth gag, a self-retaining retractor, maintains mouth opening and exposes the oropharynx. It includes a blade that depresses the tongue downward and forward, a central groove to accommodate the endotracheal tube, a mouth gag to keep the mouth open, and an anchoring system to maintain its position [2]. When connected to a suspension system, it applies sustained upward traction, optimizing visualization of the oropharynx and tonsillar fossa while exerting continuous force on oral and cervical structures. This mechanical load, in addition to surgical tissue injury, may contribute to postoperative pain, which, in children, has been reported to range from moderate to severe after adenotonsillectomy [3,4,5]. In adults, the clinical relevance of these forces has been demonstrated during suspension microlaryngoscopy, in which force metrics have been identified as predictors of tongue-related complications and opioid consumption [6]. In fact, it has been hypothesized that the force applied to the tongue during suspension microlaryngoscopy may compress the underlying neurovascular supply, particularly when a soft-tissue buffer is scarce, as observed in women [7]. In the pediatric population, where oropharyngeal soft tissue is likely reduced compared with adults [8], Boyle–Davis Gag suspension may produce similar effects. However, force metrics during Boyle–Davis Gag suspension have not been described, and their potential relationship with postoperative pain remains unknown.

Therefore, the objective of this study was to evaluate their association with force metrics and postoperative opioid consumption, pain, and emergence delirium ED and hypoactive delirium during the first postoperative hour in the post-anesthesia care unit (PACU). We also intended to describe the force metrics generated during oropharyngeal suspension for pediatric adenotonsillectomy.

We hypothesized that higher force metrics applied during oropharyngeal exposure would be associated with increased postoperative opioid consumption and pain in children undergoing adenotonsillectomy.

## 2. Materials and Methods

This is a prospective, single-blinded, observational study conducted at the Montreal Children’s Hospital and registered at ClinicalTrials.gov (NCT06115798) on 3 November 2023.

Patients younger than 18 years undergoing elective adenotonsillectomy or tonsillectomy (partial or total) at the Montreal Children’s Hospital were eligible for inclusion. Patients undergoing concurrent otorhinolaryngologic procedures (e.g., myringotomy or other airway surgery), revision procedures, or planned postoperative admission to the Pediatric Care Unit (PICU) were excluded. Patients were recruited in the preoperative area on the day of surgery. Demographic data, such as age, weight, and sex, were collected and recorded for all patients. The STBUR (Snoring, Trouble Breathing, and Un-Refreshed) questionnaire, a 5-question pediatric screening tool used by anesthesiologists to identify children at higher risk for sleep-disordered breathing, was also assessed. Additional variables collected included the surgical technique (coblation, electrocautery, or microdebrider), the level of surgical experience (trainee or attending surgeon), intraoperative anesthetic medications, and the use of a suprazygomatic maxillary nerve block for postoperative analgesia. Surgical technique, anesthetic management, and postoperative pain control were not standardized.

**Figure 1:**
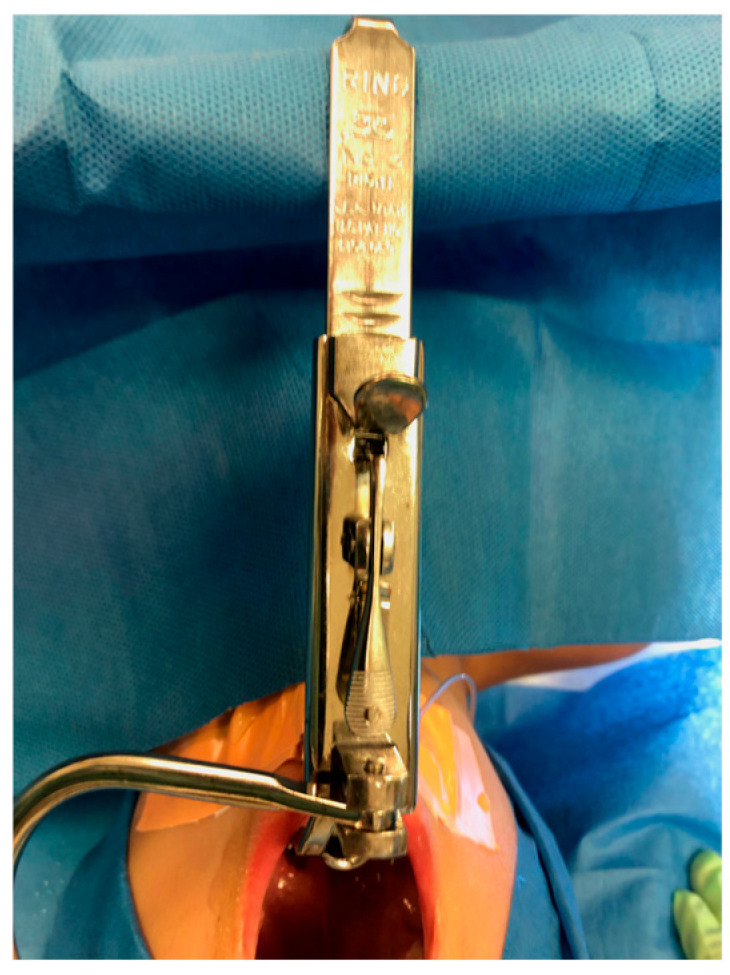
Davis–Boyle mouth gag.

**Figure 2:**
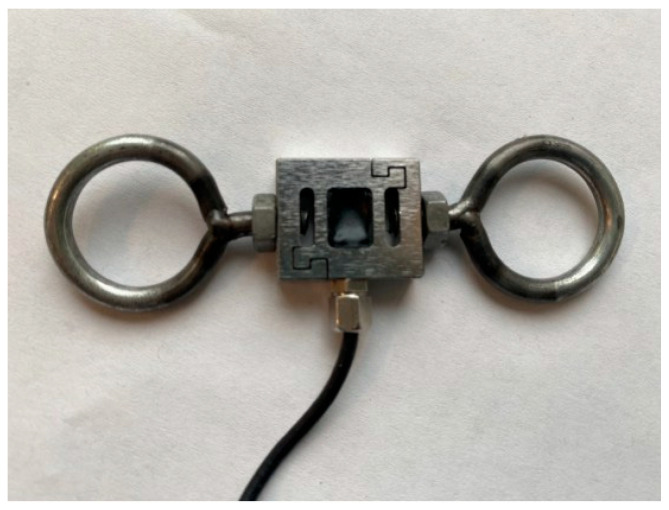
Force metric sensor. The sensor, approximately the size of a dime, was placed between the bar and the mouth gag device hook and anchored using two custom-made 4M eyebolt screws.

**Figure 3:**
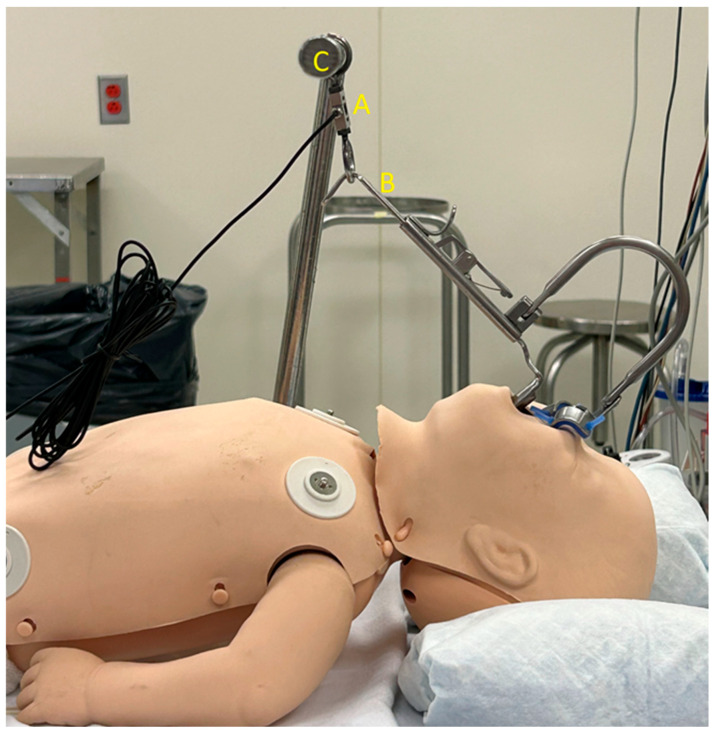
Image showing the sensor (A) anchored between the Boyle–Davis Gag (B) and the suspension bar (C). The angle between the sensor and the mouth gag was measured to calculate the effective force metrics.

To obtain a clear view and optimal access to the oropharynx, the surgeon first placed a roll under the patient’s shoulders. Then, the head was extended into the “sniffing position” and placed in a soft ring. Next, the Boyle–Davis Gag (Figure 1), a self-retaining retractor designed to maintain mouth opening and tongue depression during the procedure, was inserted. Once properly positioned, a force sensor [Force Metrics DI-1000 Load Cell Interface, Loadstar Sensors, Fremont, CA, USA, (Figure 2)] was anchored between the Boyle–Davis Gag and the suspension bar, and the angle between the sensor and the gag (Figure 3) was measured with an electronic goniometer to calculate the effective force metrics. Afterward, the ratchet mechanism was gently engaged to open the gag. Following placement, force measurements were continuously recorded throughout the procedure using custom software and a data acquisition system. The force metrics system consists of a small electronic device that connects a load cell (a sensor that measures force) to a computer via USB. The DI-1000 supplies power to the load cell and receives an electrical signal in return that varies with the applied force, which is used to estimate the force exerted on the tongue. Inside the DI-1000, a high-precision chip converts this signal into a digital value. Recorded force metrics included maximum and minimum force in Newtons (N) during the procedure, average force throughout the surgery, total suspension time in minutes, and total impulse (area under the curve of force vs. time graph) in Kilonewton-second (kN·s). Suspension time began once a non-zero recording was obtained, signifying the start of the procedure, and ended when the mouth gag device was released, marking the end of the procedure. Force signals were collected at a sampling rate of 0.01–1.0 Hz. Periods of relaxation during the procedure, when the patient was removed from suspension, were excluded from the overall suspension time and average force calculations. The surgeon was blinded to all force recordings during the perioperative period to prevent operator bias.

Postoperative pain was assessed using the Face, Legs, Activity, Cry, Consolability (FLACC) scale for children aged 7 years and younger, and the Visual Analogue Scale (VAS) for those older than 7 years. Postoperative pain was classified as mild (1–3), moderate (4–6), and severe (7–10).

Emergence delirium (ED) was assessed using the Pediatric Anesthesia Emergence Delirium (PAED) scale [9] and diagnosed when the PAED score was ≥10. We also evaluated the correlation with hypoactive delirium, a subtype characterized by reduced alertness, decreased motor activity, and social withdrawal, using the Cornell Assessment of Pediatric Delirium (CAP-D) scale, with a diagnosis made when CAP-D score was ≥9 [10].

Intraoperative medications were recorded, including both opioid and non-opioid agents. Medications administered in the PACU were also documented. Since some children were scheduled for prolonged PACU observation according to institutional protocols (e.g., patients younger than 3 years old), postoperative data collection was limited to the first hour after awakening.

Data was assessed for normality with a Shapiro–Wilk test and logarithmically transformed as needed. Continuous data that followed a normal distribution are reported as mean (SD), or median (IQR) if they were not normally distributed. Categorical data are presented as frequency (%).

Pearson’s correlation coefficient was used to assess the relationship between force measurements and opioid requirements, pain scores, presence of ED, and hypoactive delirium.

An analysis of variance was used to assess the association between force metrics and postoperative opioid requirements, postoperative pain, surgical procedures (adenotonsillectomy vs. adenoidectomy only and partial vs. total adenotonsillectomy, respectively), and surgeons’ level of experience (attending vs. trainee). A mixed-effects regression model was used to evaluate associations between force metrics and procedure type, and between surgeons’ experience levels, including an interaction term, with random intercepts for surgeons to account for heterogeneity in surgical and perioperative management. The models were controlled for sex, weight, and age as confounders. In the case of significant results, a post hoc analysis examining the interaction between surgeons’ experience level and surgical procedure (partial vs. total adenotonsillectomy) was conducted using a multivariate regression model. Subsequently, mixed-effects models were used to estimate marginal effects (95% CI).

A *p*-value of <0.05 was considered statistically significant.

Based on previous investigations [6], a minimum of 48 patients would be required to detect a correlation of 0.5 between suspension time and postoperative opioid consumption, assuming an alpha of 0.05, a power of 0.9, and a dropout rate of 20%. Statistical analysis was performed using SPSS V29 (IBM Statistics, Armonk, NY, USA).

## 3. Results

A total of 48 patients scheduled for tonsillectomy with or without adenoidectomy were enrolled in the study. Four patients were excluded due to improper calibration, and in one case, the sensor was not properly connected, resulting in a final cohort of 43 patients for analysis. Patient demographic and surgical characteristics are summarized in Table 1. Twenty-seven patients (62.8%) were male. The median age was 60 months (46 to 27), and the median weight was 20 kg (15 to 27). Surgical procedures included 38 adenotonsillectomies (88.4%), 4 adenoidectomies (9.3%), and 1 tonsillectomy (2.3%). Surgical techniques included coblation in 5 patients (11.6%), electrocautery in 22 patients (51.2%), and microdebrider use in 16 patients (37.2%). Most procedures (72.1%) were performed by attending surgeons. None of the patients received intraoperative local anesthetic injections.

The mean maximum and average forces were 29.0 N (14.8) and 11.5 N (6.8), respectively (Table 2). Mean gag suspension time was 24.6 min (12.4). The mean total impulse was 17.8 kN·s (17.1).

Thirty-three (76.7%) patients received opioids in the PACU. Regarding the primary endpoint, opioid consumption was not associated with maximum force (r = 0.16, *p* = 0.32), average force (r = 0.07, *p* = 0.65), suspension time (r = 0.18, *p* = 0.25), or total impulse (r = 0.10, *p* = 0.54). Force metrics showed no difference between patients in PACU who received opioids and those who did not (Table 3).

Postoperatively, patients reported at least one episode of mild, moderate, or severe pain in 6 (13.9%), 11 (25.6%), and 19 (44.2%) cases, respectively. Suspension time and total impulse differed according to the postoperative pain severity (Table 4).

**Figure 4:**
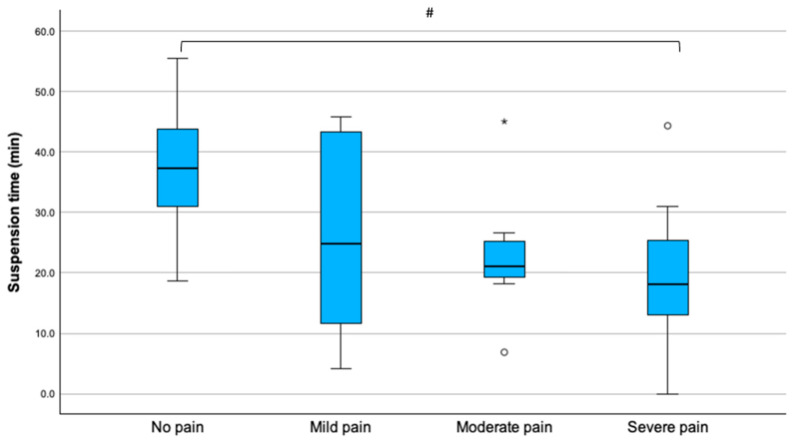
Suspension time in patients with no, mild, moderate, and severe pain. Outliers are reported as * and °. Statistical significance, ^#^ *p* = 0.022.

There was a negative correlation between pain score and suspension time (r = −0.32, *p* = 0.041), which was shorter in patients with severe pain than in those without pain (mean difference, −16.7 min; 95% CI, −31.4 to −1.9; *p* = 0.022) (Figure 4).

**Figure 5:**
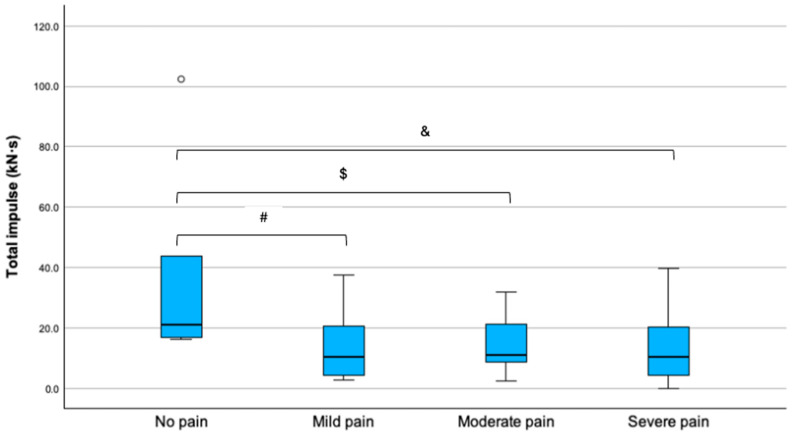
Total impulse in patients with no, mild, moderate, and severe pain. Outliers are reported as °. Statistical significance: ^#^ *p* = 0.02, ^$^ *p* = 0.011, ^&^ *p* = 0.0005.

Compared to those who did not report episodes of pain, total impulse significantly decreased in patients with mild (mean difference 2.3 kN·s; 95% CI, 3.8 to 4.2; *p* = 0.02), moderate (mean difference 2.8 kN·s; 95% CI, 5.4 to 3.9; *p* = 0.011), and severe pain (mean difference 2.3 kN·s; 95% CI, 7.6 to 3.9; *p* = 0.005) (Figure 5).

During the first hour after awakening, 26 (60.5%) and 18 (41.9%) patients had at least one episode of ED or hypoactive delirium, respectively. Nevertheless, only 10 (23.3%) patients exhibited ED without hypoactive symptoms, whereas 2 (4.6%) experienced hypoactive delirium without ED. There was no correlation between force metrics and ED or hypoactive delirium.

Suspension time decreased during the adenoidectomies compared to the tonsillectomies (Table 5). There was no difference between the procedures for average force, maximum force, and total impulse (Table 5). Similarly, there were no significant differences in force metrics between patients who underwent partial versus total tonsillectomy.

**Figure 6:**
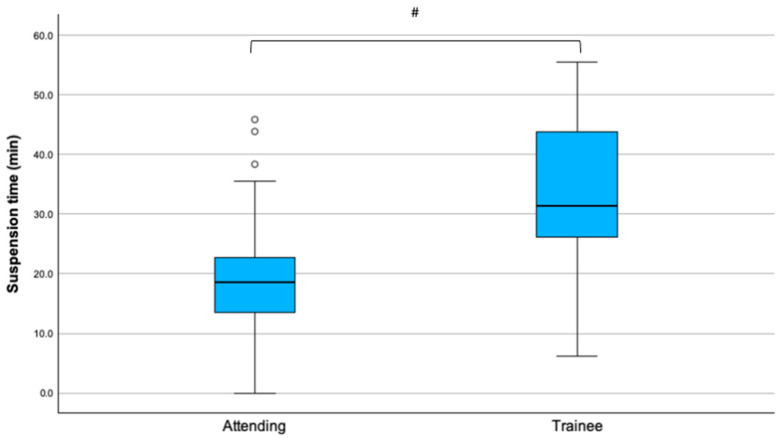
Suspension time in attending surgeons and trainees. Outliers are reported as °. Statistical significance, ^#^ *p* = 0.003.

Twelve (27.9%) surgical procedures were performed by trainees (resident or fellow). When comparing the surgeon’s level of experience, trainees had a greater suspension time compared to the attending surgeons [33.2 (12.8) vs. 21.1 (10.5) min, mean difference −12.1 (95% CI −19.9 to −4.3), *p* = 0.003 (Figure 6)]. There were no differences for maximum force, average force, and total impulse. In particular, trainees took longer to perform a total adenotonsillectomy compared to a partial one, whereas attendings did not (Table 6).

## 4. Discussion

Our study suggests that the force applied during Boyle–Davis Gag suspension does not correlate with our postoperative outcomes of interest, namely opioid consumption, pain scores, and delirium in children. To date, no other studies have investigated the force metric during oropharyngeal suspension.

The most common symptoms after tonsillectomy in children are pain, especially during swallowing, which limits the child’s ability to resume eating, drinking, and taking oral medications [3]. In adults, maximum force >133 N was predictive of postoperative complications, such as increased postoperative pain, dysgeusia, paresthesia, and paresis [7,11]. Feng et al. [6] found that suspension time was positively correlated with postoperative opioid requirements in adults undergoing suspension microlaryngoscopy. Conversely, our study found only a similar yet negative correlation between suspension time and reported pain. Moreover, both the suspension time and the total impulse were longer in a patient with no pain. Interpreting these discrepancies is difficult and highly speculative. It is possible that the higher force (and impulse) allowed for better surgical exposure and less tissue damage. These results may also be attributable to differences in surgical procedures and the relatively low force used in children. Indeed, adult suspension microlaryngoscopy is routinely employed in patients with a wide range of body mass index and for a variety of surgical interventions, including lesion excision, endoscopic oncologic surgery, and airway dilatation [6,7,11,12]. Consequently, the forces may differ tenfold, with the maximum force ranging from 45 N to 462 N and the average force ranging from 19 N to 271 N, respectively [12]. In our cohort, the maximum force reported was, on average, 75% lower than that reported in adults. Secondly, a previous investigation found no association between suspension times of less than 30 min, similar to those reported in our investigation, and postoperative complications, including pain [7]. Likewise, patients who did not experience complications exhibited a total impulse of 114 kN·s [7], which is 10 times the value reported in our investigation. In our study, extended procedures (i.e., prolonged suspension time) may also have allowed the intraoperative analgesic sufficient time to reach its peak effect before recovery in the PACU. It is noteworthy that approximately 40% of the patients were administered intraoperative morphine, which exhibits a significant peak effect approximately 45 min post-administration.

Our data also did not find an association between force metric and ED or hypoactive delirium. Emergence delirium is a well-recognized early postoperative disturbance in children, characterized by altered awareness, disorientation, and perceptual disturbances following general anesthesia. It is particularly prevalent in pediatric patients, with an incidence ranging from 25% to 80%, typically occurring within the first 30 min after anesthesia [9]. Otolaryngologic procedures have been associated with an increased risk of ED [13]. Disruptive behaviour in the PACU is associated with increased nursing workload and staffing requirements. It also negatively affects healthcare team functioning and parents’ satisfaction with the quality of the child’s recovery [14,15]. Moreover, children experiencing ED are at greater risk for negative postoperative behavioural changes [16]. It has been reported that as many as 15% of children may experience both ED and pain, with those showing ED being more prone to postoperative pain [17,18]. In our cohort, the incidence of ED was comparable to that reported in previous studies of children. Of interest, 40% of children experiencing ED also showed signs of hypoactive delirium. This incidence is 1.5 times higher than reported in a previous investigation [10] but similar to that reported in the PICU [19]. Compared to children with ED, children experiencing hypoactive delirium present as quiet, confused, and disoriented. They do not establish eye contact with caregivers and may be unaware of their surroundings. Instead of exhibiting restlessness, agitation, and purposeless movements, these children display minimal movement when awake, are non-communicative, and do not respond to social interactions. Hypoactive delirium has been generally underreported and confined to investigations within intensive care settings [19]. The difference between our results and those reported by Lee-Archer et al. [10] may be attributable to differences in patient populations, with our cohort being younger and limited to adenotonsillectomy procedures, which are known to carry a high incidence of delirium and pain upon awakening [20,21].

Partial tonsillectomy showed force metrics similar to those of total adenoidectomy, except for suspension time. Current evidence shows that partial adenotonsillectomy is associated with reduced postoperative pain [22]. As the force metrics did not differ significantly across the different surgical techniques, our investigation supports that this surgical technique is associated with less postoperative pain due to less tissue trauma [22].

Finally, our findings showed that only the suspension time differed significantly between attendings and trainees, with the latter taking longer to perform the total adenotonsillectomy, consistent with their lower level of expertise. In this context, increased postoperative pain may be attributable to the less refined surgical technique of trainees rather than to force metrics per se.

In adult patients, it has been hypothesized that active use of large-force metric monitoring during suspension microlaryngoscopy may reduce exertion throughout the procedure, thereby potentially decreasing postoperative pain and complications [11,23]. Although our data do not support routine use of force metrics during Boyle–Davis Gag suspension in children, the ability to objectively measure and modulate suspension-related forces may have important implications for clinical practice. This may be especially relevant for populations at increased risk of cervical instability, such as children with Trisomy 21, in whom up to 30% may have atlantoaxial or atlanto-occipital instability, placing them at increased risk of spinal cord injury during suspension [24]. Although 5% of children undergoing adenotonsillectomy have Trisomy 21, there are no universal guidelines for managing these patients during suspension for adenotonsillectomy, yet the number of reported cases of spinal cord injury is relatively small [25]. In our study, the maximum force reported may be below the threshold for spinal cord injury [26], which may help justify the low incidence of spinal cord injury during adenotonsillectomy in the patient population.

### Limitations

This study has several limitations that should be acknowledged.

First, the sample size was small, which limits the reliability of the multivariate logistic regression analysis.

Second, our outcome assessment was limited to the first postoperative hour. However, a previous investigation showed that maximum force may be predictive of postoperative symptoms for up to two weeks after surgery in adults [11].

Third, our investigation did not include patients with atlantoaxial instability, a population that may benefit most from measuring the force metric during Boyle–Davis Gag suspension.

## 5. Conclusions

This study introduces innovative force measurements of Boyle–Davis Gag suspension during adenotonsillectomy in the pediatric population and provides initial reference values for applied force. No association was observed between force magnitude and immediate postoperative opioid consumption, emergence delirium, or hypoactive delirium, regardless of surgical technique. Suspension time and total impulse may be associated with reported pain, but the clinical significance of this finding remains unclear.

Future analyses with larger cohorts and standardized perioperative management may help define clinically relevant force thresholds and further clarify the role of suspension-related forces in pediatric perioperative outcomes.

## Figures and Tables

**Table 1:** Patient variables of children undergoing gag suspension for tonsillectomy and/or adenoidectomy (N = 43).

Patient Characteristics	N = 43
Sex	
Male	27 (62.8%)
Female	16 (37.2%)
Age (Months)	
Mean (SD)	67.3 (34.6)
Median (IQR)	60.0 (46.0 to 27.0)
Weight (kg)	
Mean (SD)	25.0 (14.0)
Median (IQR)	20.0 (15.2 to 27.0)
Procedures	
Adenotonsillectomy	38 (88.4%)
Tonsillectomy	1 (2.3%)
Adenoidectomy	4 (9.3%)
Tonsillectomy	
Partial	17 (39.5%)
Total	22 (51.2%)
Surgical approach	
Coablation	5 (11.6%)
Electrocautory	22 (51.2%)
Microdebride	16 (37.2%)
STUBR score	
Mean (SD)	2.3 (1.6) (CI 1.8–2.9)
Median (IQR)	2 (1.0 to 3.5)

Data presented as mean (SD), median (IQR), and number (%). STUBR: Snoring, Trouble Breathing, and Un-Refreshed.

**Table 2:** Description of force metrics.

Variable	Mean (SD)	Median (IQR)	Missing Values
Maximum force (N)	29.0 (14.8)	26.0 (15.6 to 40.4)	2
Average force (N)	11.5 (6.8)	10.2 (7.9 to 13.5)	None
Suspension time (min)	24.6 (12.4)	22.0 (16.6 to 31.4)	2
Total impulse (kN·s)	17.8 (17.1)	14.1 (6.9.2 to 21.1)	None

Data presented as mean (SD) and median (IQR).

**Table 3:** Comparison of force metrics between patients who received opioids in the Post-Anesthesia Care Unit and those who did not.

Variable	No Opioids (n = 10) Mean (SD)	Opioids (n = 33) Mean (SD)	Mean Difference (95% CI)	*p*-Value
	
Maximum force (N)	24.3 (19.3)	30.4 (13.2)	−6.0 (−18.8 to 4.7)	0.26
Average force (N)	12.1 (11.2)	11.3 (4.9)	−0.8 (−5.9 to 4.2)	0.74
Suspension time (min)	25.7 (13.5)	24.3 (12.2)	−1.4 (−10.6 to 7.8)	0.76
Total impulse (kN·s)	20.4 (29.7)	17.0 (11.2)	−3.4 (−16.1 to 9.3)	0.59

Data are presented as mean and standard deviation (SD). Mean difference and 95% confidence intervals (CI) were calculated between groups.

**Table 4:** Analysis of variance on the association of force metrics and pain severity.

Variable	No Pain (n = 6)	Mild Pain (n = 6)	Moderate Pain (n = 11)	Severe Pain (n = 19)	*p*-Value
Maximum force (N)	32.5 (20.8)	20.7 (11.2)	27.9 (12.7)	31.0 (15.3)	0.48
Average force (N)	16.4 (12.7)	9.9 (5.2)	11.0 (6.4)	10.8 (4.5)	0.33
Suspension time (min)	37.2 (12.4)	25.8 (17.9)	22.7 (9.1)	20.7 (10.0)	0.035 *
Total impulse (kN·s)	36.9 (33.6)	14.4 (13.0)	15.1 (10.3)	14.0 (9.9)	0.029 *

Data are presented as mean and standard deviation (SD). * Statistical significance, *p* < 0.05.

**Table 5:** Comparison of force metrics between patients who underwent adenoidectomy only and those who underwent adenotonsillectomy.

Variable	Adenoidectomy Only (n = 4)	Adenotonsillectomy (n = 39)	Mean Difference (95% CI)	*p*-Value
Maximum force (N)	18.6 (14.5)	30.0 (14.6)	11.5 (−4.0 to 27.0)	0.14
Average force (N)	9.1 (5.7)	11.8 (6.9)	2.7 (−4.6 to 10.0)	0.45
Suspension time (min)	10.7 (10.1)	26.1 (11.7)	15.4 (3.0 to 27.8)	0.016 *
Total impulse (kN·s)	5.0 (4.2)	19.2 (17.4)	14.2 (−3.7 to 32.1)	0.12

Values are reported as mean and standard deviation (SD). Mean differences and corresponding 95% confidence intervals (CI) between groups are presented. * Statistically significant difference, *p* < 0.05.

**Table 6:** Post hoc analysis of the procedure x level interaction.

Variable	Partial Adenotonsillectomy	Total Adenotonsillectomy	Mean Difference (95% CI)	Adjusted *p*-Value
Attending	24.9 (3.7)	20.7 (3.0)	−4.2 (−13.3 to 4.8)	0.35
Trainee	34.0 (3.8)	48.5 (5.7)	14.5 (1.4 to 27.6)	0.031 *

Values are reported as mean (SD) and the mean differences and corresponding 95% CI between Adenoidectomy and Adenotonsillectomy. * Statistically significant difference, *p* < 0.05.

## Data Availability

The original contributions presented in the study are included in the article, further inquiries can be directed to the corresponding author.
